# Beyond lithium-ion batteries: what's powering tomorrow's breakthroughs?

**DOI:** 10.1093/nsr/nwaf287

**Published:** 2025-07-15

**Authors:** Qiang Zhang, Lianzhou Wang

**Affiliations:** Beijing Key Laboratory of Complex Solid State Batteries & Tsinghua Center for Green Chemical Engineering Electrification, Department of Chemical Engineering, Tsinghua University, China; State Key Laboratory of Chemical Engineering and Low-Carbon Technology, Tsinghua University, China; Australian Institute for Bioengineering and Nanotechnology and School of Chemical Engineering, The University of Queensland, Australia; Department of Applied Biology and Chemical Technology, The Hong Kong Polytechnic University, China

Electrochemical energy storage systems have undergone remarkable evolution since the earliest observed manifestations of galvanic phenomena. Batteries, as electrochemical energy conversion devices, operate through controlled redox reactions that transform stored chemical energy into electrical energy with high efficiency. These systems now permeate our current society, enabling diverse applications ranging from miniaturized medical implants to grid-scale renewable energy integration.

The historical trajectory of battery technology reveals a fascinating interplay between fundamental discoveries and engineering innovations. Archaeological evidence suggests primitive electrochemical cells may have existed as early as the Parthian period (250 BCE–224 CE), exemplified by the controversial Baghdad artifact comprising an iron–copper arrangement in an acidic medium. The scientific foundation for modern batteries emerged in the late 18th century through Galvani's seminal work on bioelectrical phenomena, and Volta's subsequent development of the first true electrochemical cell—the voltaic pile (1800). While revolutionary for its time, this zinc–copper system suffered from significant polarization effects and electrolyte depletion.

Subsequent advancements addressed these limitations through systematic materials innovation and system engineering. Daniell's cell (1836) introduced ion-selective separation, while Planté’s lead–acid system (1859) established the first practical rechargeable architecture. The late 19th century witnessed crucial transitions from liquid to quasi-solid electrolytes (Gassner's dry cell, 1886) and the development of alternative chemistries (Jungner's Ni–Cd system, 1899). The mid-20th century brought Urry's alkaline battery (1950s), which dramatically improved energy density and shelf life for primary cells.

The modern era of energy storage dawned with investigations into lithium-based systems during the 1970s' energy crisis. Whittingham's pioneering work at Exxon demonstrated the exceptional theoretical capacity of lithium intercalation compounds, though safety concerns regarding metallic lithium anodes persisted. Goodenough's identification of lithium cobalt oxide (1980) as a stable high-potential cathode material represented a watershed moment, effectively doubling available cell voltages. Yoshino's subsequent replacement of lithium metal with carbonaceous anodes (1985) resolved critical safety issues, culminating in Sony's successful commercialization of lithium-ion technology (1991).

Today's lithium-ion batteries represent the pinnacle of electrochemical engineering, achieving remarkable energy densities (>180 Wh/kg) and cycle lives (>1000 cycles). However, emerging challenges in resource sustainability, safety and performance demands necessitate exploration beyond conventional lithium-ion paradigms. This Special Topic highlights cutting-edge research in next-generation energy storage systems, including:

- alternative cation systems (Na^+^, K^+^, Zn^2+^, Al^3+^);
- solid-state architectures and related all-solid-state batteries;
- advanced electrolyte engineering;
- novel electrode materials and interfacial engineering.

The collection comprises two perspectives, nine original research articles, two comprehensive reviews and an expert interview, collectively addressing critical challenges in post-lithium-ion battery development.

**Perspectives:** Chen *et al.* provide fundamental insights into non-aqueous electrolyte behavior, contrasting classical solution models with battery-relevant phenomena [1]. Zhao and colleagues examine proton intercalation chemistry, revealing new opportunities for aqueous energy storage [2].

**Notable research advances include:**

- Shao and co-workers’ innovative metal phosphate electrode design [3];
- Wu and co-workers’ fluorine-doped solid electrolytes for high-voltage operation [4];
- Zhou and co-workers’ mechanistic study of lithium plating failure modes [5];
- He and co-workers’ oxygen-concentrated electrolytes for Li–O_2_ batteries [6];
- Xing and co-workers’ prelithiated silicon using lithium naphthalenide [7];
- Su and co-workers’ finding on a chemically separated two-phase cathode structure [8];
- Pang and co-workers’ dendrite-free 3D Al with ultra-long cycling for aluminum batteries [9];
- Wang and co-workers’ single-atom catalysts for electron transportation in room-temperature sodium–sulfur batteries [10];
- Hu and co-workers’ graphitic nitrogen adjacent to the Fe–N_4_ centers for Zn–air batteries with high peak power density [11].

**Comprehensive reviews:** Fan *et al.* critically analyze aqueous zinc–halogen systems [12], while Luo's team assesses iron-based flow battery advancements [13].

**Expert insights:** Our interview with Professor Zaiping Guo [14] explores future directions in sustainable, high-performance energy storage.

We extend our deepest gratitude to all contributors and reviewers whose expertise and effort have shaped this timely collection. As we stand at the threshold of a new energy storage paradigm, these works collectively illuminate pathways toward safer, more sustainable and higher-performance electrochemical systems for our energy-intensive future.
